# Neuronal surface antigen-specific immunostaining pattern on a rat brain immunohistochemistry in autoimmune encephalitis

**DOI:** 10.3389/fimmu.2022.1066830

**Published:** 2023-01-16

**Authors:** Naomi Nagata, Naomi Kanazawa, Tomomi Mitsuhata, Masaki Iizuka, Makoto Nagashima, Masaaki Nakamura, Juntaro Kaneko, Eiji Kitamura, Kazutoshi Nishiyama, Takahiro Iizuka

**Affiliations:** Department of Neurology, Kitasato University School of Medicine, Sagamihara, Japan

**Keywords:** autoimmune encephalitis, immunohistochemistry, autoantibodies, neuronal surface antigens, tissue-based assay, cell-based assay

## Abstract

A variety of neuronal surface (NS) antibodies (NS-Ab) have been identified in autoimmune encephalitis (AE). Tissue-based assay (TBA) using a rodent brain immunohistochemistry (IHC) is used to screen NS-Ab, while cell-based assay (CBA) to determine NS antigens. Commercial rat brain IHC is currently available but its clinical relevance remains unclear. Immunostaining patterns of NS antigens have not been extensively studied yet. To address these issues, we assessed a predictive value of “neuropil pattern” and “GFAP pattern” on commercial IHC in 261 patients, and characterized an immunostaining pattern of 7 NS antigens (NMDAR, LGI1, GABAaR, GABAbR, AMPAR, Caspr2, GluK2). Sensitivity and specificity of “neuropil pattern” for predicting NS-Ab were 66.0% (95% CI 55.7-75.3), and 98.2% (95% CI 94.8-99.6), respectively. False-positive rate was 1.8% (3/164) while false-negative rate was 34.0% (33/97). In all 3 false-positive patients, neuropil-like staining was attributed to high titers of GAD65-Ab. In 33 false-negative patients, NMDAR was most frequently identified (n=18 [54.5%], 16/18 [88.9%] had low titers [< 1:32]), followed by GABAaR (n=5). Of 261 patients, 25 (9.6%) had either GFAP (n=21) or GFAP-mimicking pattern (n=4). GFAP-Ab were identified in 21 of 31 patients examined with CBA (20 with GFAP pattern, 1 with GFAP-mimicking pattern). Immunostaining pattern of each NS antigen was as follows: 1) NMDAR revealed homogenous reactivity in the dentate gyrus molecular layer (DG-ML) with less intense dot-like reactivity in the cerebellar granular layer (CB-GL); 2) both GABAaR and GluK2 revealed intense dot-like reactivity in the CB-GL, but GABAaR revealed homogenous reactivity in the DG-ML while GluK2 revealed intense reactivity along the inner layer of the DG-ML; and 3) LGI1, Caspr2, GABAbR, and AMPAR revealed intense reactivity in the cerebellar ML (CB-ML) but LGI1 revealed intense reactivity along the middle layer of the DG-ML. Whereas, Caspr2, GABAbR, and AMPAR revealed similar reactivity in the DG-ML but some difference in other regions. TBA is useful not only for screening NS- or GFAP-Ab but also for estimating NS antigens; however, negative results should be interpreted cautiously because “neuropil pattern” may be missed on commercial IHC when antibody titers are low. Antigen-specific immunoreactivity is a useful biomarker of AE.

## 1 Introduction

Autoimmune encephalitis (AE) is a form of encephalitis that occurs as a result of a brain-specific immune response and usually associates with an antibody against a neuronal or glial, cell surface antigen (1). A variety of neuronal surface (NS) antibodies (NS-Ab) have been identified in patients with AE or related disorder (2). Glial fibrillary acidic protein (GFAP) antibodies (GFAP-Ab) have also been reported concurrently with NMDA receptor (NMDAR) antibodies (NMDAR-Ab) without association with distinct clinico-radiologic features (3). Tissue-based assay (TBA) using a rodent brain immunohistochemistry (IHC) adapted to NS antigens is used to screen NS-Ab, while cell-based assay (CBA) using human embryonic kidney (HEK) 293 cells expressing target antigens on the cell surface membrane is used to determine the NS antigens (4). Live hippocampal neuronal cultures are also used to confirm the presence of NS-Ab. However, these studies are mainly performed at research laboratories (1).

In clinical practice; however, it is difficult to measure all NS-Ab identified to date. A commercial fixed CBA is currently available for many but not for glycine receptor (GlyR), γ-aminobutyric acid A receptor (GABAaR), or glutamate kainate receptor subunit 2 (GluK2), and limitations of the commercial CBA as a diagnostic test of AE have been reported (5). It is an issue which NS antigen should be examined with CBA in the individual cases. Immunostaining patterns highly characteristic of NS antigens have been described (3) but not extensively studied yet. A commercial IHC is also currently available but its clinical relevance remains unclear. If TBA reveals individual NS-antigen-specific immunostaining pattern, it will facilitate identification of the target antigen by choosing appropriate CBA, ultimately leading to early diagnosis and early initiation of appropriate treatment.

To address these issues, we conducted this study to clarify 1) whether the commercial IHC is clinically useful for screening NS- or GFAP-Ab (Part I), and 2) whether TBA reveals an immunostaining pattern highly characteristic of major individual NS antigens (Part II).

## 2 Materials and methods

### 2.1 Patient selection and antibody measurement

First, we retrospectively reviewed the clinical information of 622 patients with suspected AE or related disorder, who underwent a testing for NS-Ab between January 1, 2007 and October 31, 2022. These patients were admitted to Kitasato University Hospital or other hospitals between January 1, 1999 and September 30, 2022 with suspected AE or related disorder; in 7 patients who were admitted to Kitasato University Hospital before January 1, 2007, archived cerebrospinal fluid (CSF)/sera obtained at the onset of disease were used for antibody assays. The CSF/sera obtained from 388 patients (62.4%) of this cohort were referred from other 145 hospitals widely distributed in Japan to Kitasato University to examine NS-Ab. The detailed clinical information was provided from each physician to TI (a primary investigator of the study).

The inclusion criteria of the patients who underwent a testing for NS-Ab are as follows: 1) AE or related neurological disorder is highly suspected based on clinical assessment; 2) detailed clinical information that supports the clinical diagnosis is available for review by TI, including clinical course from the onset of symptoms or prodromal viral-like illness, past history, family history, a habit of smoking or drinking, regular medications, neuropsychological assessment on admission, laboratory test results [blood, CSF, brain or spinal MRIs, body CT, electroencephalography, needle or surface electromyography (when suspected of having stiff-person spectrum disorder, Morvan syndrome, or Isaacs’ syndrome)], and subsequent course of the disease when available; and 3) written informed consent is obtained from the patients or their proxies. When all the criteria were fulfilled, we accepted measurement of NS-Ab. However, reasonable exclusion of alternative cause was not considered mandatory at the time of collection of sera/CSF samples. Therefore, we included patients in whom alternative cause had not been completely excluded yet. The sera/CSF samples mainly obtained at the acute phases and kept frozen until antibody testing.

NS-Ab were measured at the laboratory of Josep Dalmau (University of Pennsylvania, Philadelphia, or IDIBAPS Hospital Clínic, Barcelona) with a rat brain in-house IHC adapted to NS antigens and CBA (6–14); they included antibodies against the NMDAR, α-amino-3-hydroxy-5-methyl-4-isoxazolepropionic acid receptor (AMPAR), GABAaR, γ-aminobutyric acid B receptor (GABAbR), metabotropic glutamate receptor 5 (mGluR5), metabotropic glutamate receptor 1 (mGluR1), dipeptidyl peptidase-like protein 6 (DPPX), contactin-associated protein-like 2 (Caspr2), leucine-rich glioma-inactivated 1 (LGI1), neurexin 3, GlyR or GluK2. Both serum and CSF were examined in all patients except 9 (only CSF [n=5] or serum [n=4] was available). These NS antigens were measured with CBA mainly based on the clinical phenotypes of each patient and/or immunostaining pattern on in-house IHC performed at the laboratory of Josep Dalmau.

In addition to NS-Ab, myelin oligodendrocyte glycoprotein (MOG) and aquaporin-4 (AQP4) antibodies were examined with CBA in patients with overlapping encephalitis and demyelinating syndrome (15). GFAP-Ab were also subsequently examined in CSF at the laboratory of Josep Dalmau with established CBA (3) in patients who were clinically suspected of having GFAP-positive meningoencephalomyelitis or when TBA performed at Kitasato University revealed immunoreactivity suggesting GFAP. Autoantibodies against classical paraneoplastic intracellular antigens (CV2/CRMP5, Ma2, Ri, Yo, Hu, amphiphysin) were measured in serum at Kitasato University or referring hospital with immunoblot (Euroimmun AG), when clinically considered necessary. Glutamate decarboxylase (GAD) antibodies (GAD-Ab) were also measured in serum with either radioimmunoassay, enzyme-linked immunosorbent assay (ELISA), or enzyme immunoassay (EIA).

**Figure 1:**
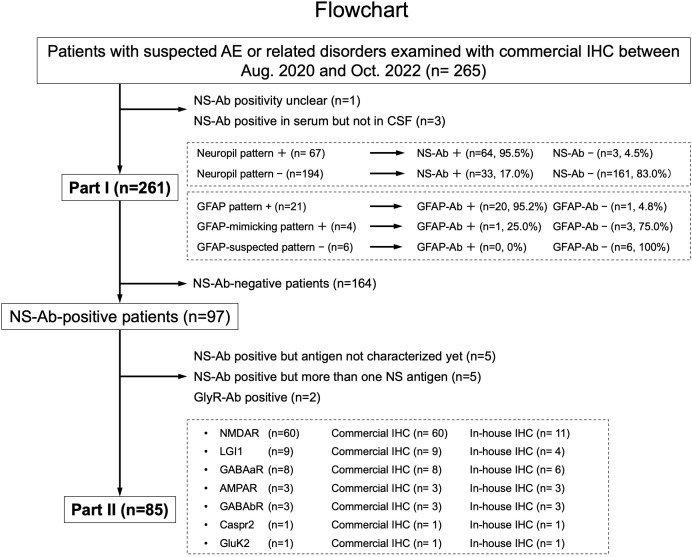
This figure shows a diagram of the study conducted including Part I and Part II. In Part I, a predictive value of a “neuropil pattern” suggesting NS-Ab and a pattern suggesting GFAP-Ab were mainly investigated, whereas in Part II, an immunostaining pattern of the pre-selected 7 NS antigens are evaluated based on the subjects listed here. The number of patients is shown in parentheses. AE, autoimmune encephalitis; Ab, antibodies; GFAP, glial fibrillary acidic protein; IHC, immunohistochemistry; NS, neuronal surface.

Next, we selected 265 patients (42.6%), whose archived CSF was examined with commercial IHC at Kitasato University between August 1, 2020 and October 31, 2022 to evaluate an immunostaining pattern, irrespective of NS-Ab-positivity. In this study, the results of IHC using sera were not included in the evaluation of immunostaining pattern because it is sometimes difficult to evaluate NS-antigen-specific pattern due to concurrent high background reactivity, intravenous immunoglobulins administered before the collection of the sera, or concurrent antibodies to intracellular antigens, of which clinical relevance in AE is unclear. After exclusion of 4 patients (see **Figure 1**), 261 (152 female 58.2%), median age at onset 46 years [range, 5-91 years]) were included in Part I. We also performed in-house IHC adapted to NS antigens in 54 patients to compare immunostaining pattern between in-house and commercial IHC.

In Part II, among NS antigens identified in the CSF, we selected 7 major antigens (NMDAR, AMPAR, GABAaR, GABAbR, LGI1, Caspr2, and GluK2). The GlyR was not included because it is difficult to see the reactivity with GlyR on the hippocampus or cerebellum section (1). The DPPX was also not included because no DPPX antibodies were identified in our cohort. After exclusion of 164 NS-Ab-negative patients and 12 NS-Ab-positive patients (5 with antibodies against NS antigen not characterized yet, 5 with antibodies against more than one NS antigen, 2 with GlyR antibodies [GlyR-Ab]), 85 were finally selected for Part II. An immunostaining pattern was evaluated with both in-house and commercial IHC in each group of NS antigen (**Figure 1**).

### 2.2 In-house and commercial rat brain IHC

**Figure 2:**
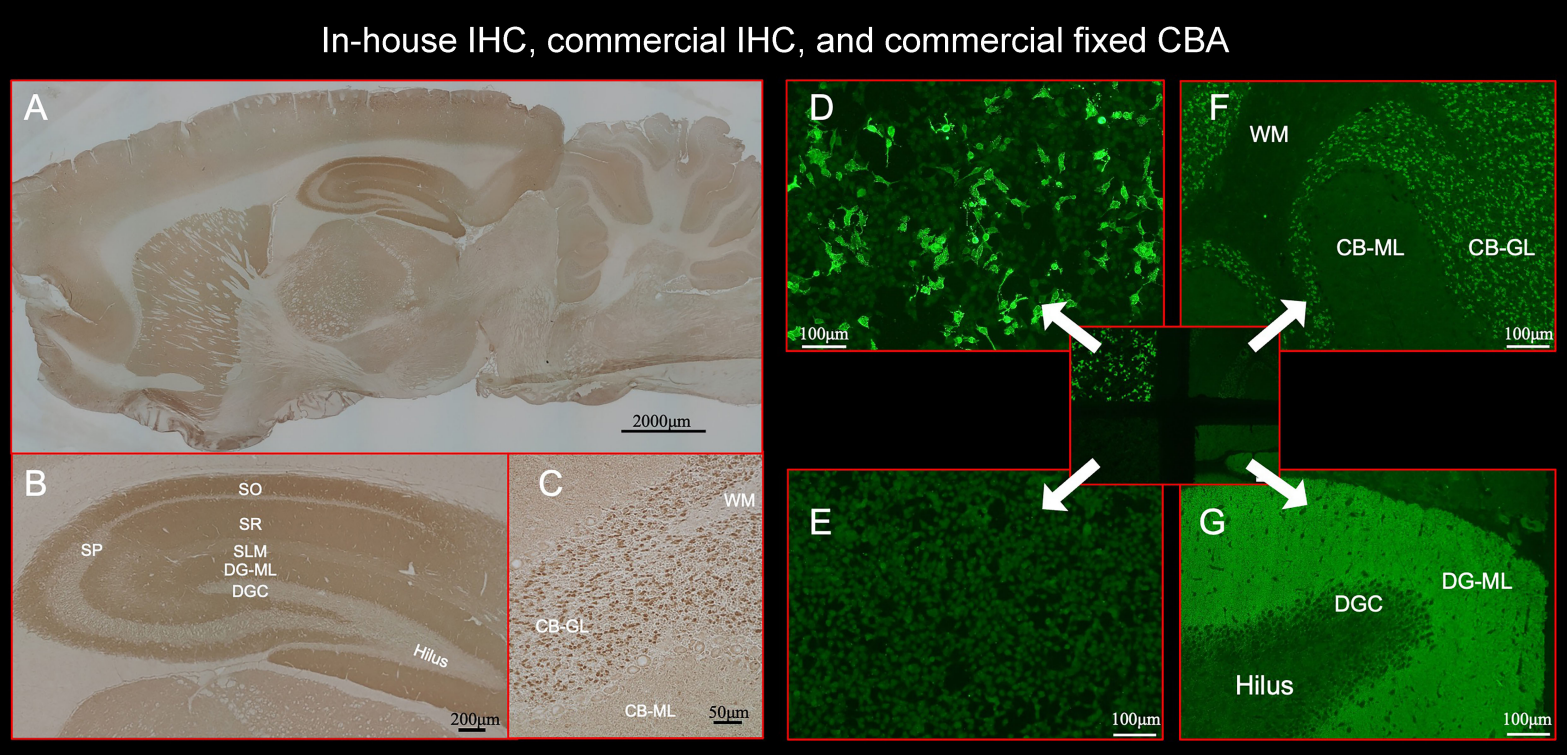
This figure shows the results of immunostaining pattern using in-house IHC **(A–C)** and commercial kit, which consists of 4 biochips per field containing the NMDAR-transfected cells **(D)**, control-transfected cells **(E)**, cerebellum **(F)**, and hippocampus **(G)**. Note intense reactivity with NS antigen in the DG-ML **(A, B, G)**, less intense dot-like reactivity in the CB-GL but no apparent reactivity on the CB-ML **(C, F)**. This staining pattern is consistent with NMDAR. **(D)** shows intense staining on NMDAR-transfected cells but not on control-transfected cells **(E)**, confirming the diagnosis of anti-NMDAR encephalitis. Antibody assay was performed using CSF (diluted 1:2) obtained from a patient with anti-NMDAR encephalitis (antibody titer 1:2048), with in-house IHC adapted to NS antigens **(A–C)**, commercial fixed CBA **(D, E)**, and commercial IHC **(F, G)**. See Text. CBA, cell-based assay; CB-GL, cerebellar granular layer; CB-ML, cerebellar molecular layer; DGC, dentate granule cells; DG-ML, dentate gyrus molecular layer; IHC, immunohistochemistry; NMDAR, NMDA receptor; NS, neuronal surface; SLM, stratum lacunosum moleculare; SO, stratum oriens; SP, stratum pyramidale; SR, stratum radiatum; WM, white matter.

In-house IHC was performed at Kitasato University with patients’ CSF (diluted 1:2) using a standard technique (16, 17). In brief, Wistar female rats at 10 weeks of age were anesthetized and euthanized by decapitation without tissue perfusion. Brains were removed, fixed in 4% paraformaldehyde for 1 hour at 4°C, cryoprotected in 40% sucrose for 48 hours at 4°C, embedded in freezing media, and snap frozen in isopentane chilled with liquid nitrogen. Ten micron-thick sections were incubated with 0.3% H_2_O_2_ and blocked with 10% goat serum, and then incubated with patient’s CSF (diluted 1:2) overnight at 4°C. The sections were incubated with a secondary biotinylated goat anti-human IgG for 1 hour, and the reactivity developed with the avidin-biotin-peroxidase method (**Figures 2A–C**). The animal study was reviewed and approved by the Animal Experimentation and Ethics Committee of the Kitasato University School of Medicine (2022-031) and were performed in accordance with institutional guidelines for animal experimentation, which are based on the Guidelines for Proper Conduct of Animal Experiments published by the Science Council of Japan.

Commercial IHC was also performed at Kitasato University using a kit (Euroimmun AG, product No: FA 111m-3) following the instruction of the company with indirect immunofluorescent assay (IIFA). The kit consists of 4 biochips per field containing the NMDAR-transfected cells (**Figure 2D**), control-transfected cells (**Figure 2E**), cerebellum (**Figure 2F**), and hippocampus (**Figure 2G**). IIFA was evaluated with an Olympus BX53 fluorescence microscope (Olympus, Japan). In this study, we evaluated an immunostaining pattern using CSF (diluted 1:2) that was used in in-house IHC.

### 2.3 Evaluation of “neuropil pattern” on commercial rat brain IHC

In Part I, we assessed a predictive value of a pattern of neuropil reactivity (“neuropil pattern”) suggesting the presence of NS-Ab. In this study, the “neuropil pattern” was considered positive when apparent reactivity with NS antigens was visually identified on commercial IHC at the level of hippocampus and cerebellum section. Immunostaining pattern was initially assessed independently by 3 observers (NK, NN, and TI), and only patients whose CSF samples (diluted 1:2) were finally judged to be positive by all of them were considered positive with neuropil pattern. A pattern suggesting reactivity with glial surface antigens (AQP4 or MOG) was not included in the “neuropil pattern”. The NS-Ab-positivity or negativity was determined based on the final report from the laboratory of Josep Dalmau. Then, we determined sensitivity and specificity of the “neuropil pattern” for predicting NS-Ab as well as false-negative (NS-Ab positive but “neuropil pattern” negative) or false-positive rate (NS-Ab negative but “neuropil pattern” positive). In Part I, we did not exclude patients with autoantibodies against multiple NS antigens or antigens not characterized yet.

To assess factors potentially associated with false-negative results, we measured antibody titers in CSF using fixed CBA (Euroimmun AG) in patients with NMDAR-Ab because NMDAR was most frequently identified in the false-negative patients. We also performed in-house IHC in some of the patients to evaluate whether in-house IHC can reveal the “neuropil pattern” in the false-negative patients.

### 2.4 Evaluation of “GFAP pattern” on commercial rat brain IHC

**Figure 3:**
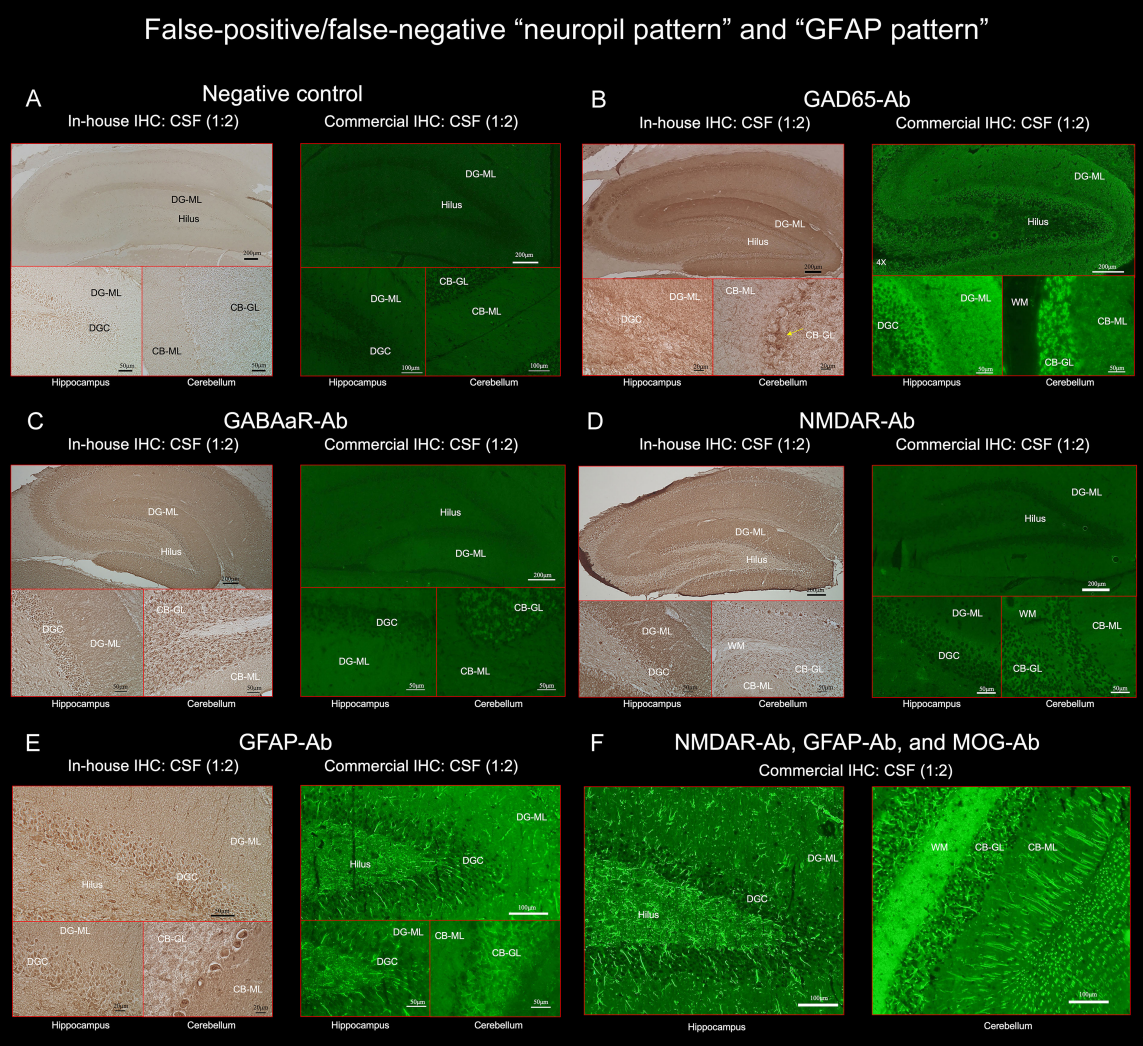
**(A)** does not show apparent “neuropil pattern” with negative control, but **(B)** reveals some reactivity mimicking “neuropil pattern” in the middle and outer layers of the DG-ML. Reactivity in the surroundings of the DGC, Purkinje cells, and CB-GL are consistent with a pattern of GAD65 reactivity. **(C, D)** did not reveal apparent “neuropil pattern” on commercial IHC but visually recognizable reactivity on in-house IHC. **(E, F)** reveal a cactus thorn-like or filamentous staining consistent with a pattern of GFAP reactivity. Note that GFAP pattern is more clearly shown on commercial IHC than on in-house IHC **(E)**. **(F)** shows intense reactivity with GFAP and MOG but not apparent “neuropil pattern”. CB-GL, cerebellar granular layer; CB-ML, cerebellar molecular layer; DGC, dentate granule cells; DG-ML, dentate gyrus molecular layer; WM, white matter.

In Part I, we also assessed a predictive value of a pattern suggesting the presence of GFAP-Ab. We considered “GFAP-suspected”, when either of the following patterns was seen: 1) “GFAP pattern”, which is a pattern of GFAP reactivity showing a cactus thorn-like or filamentous reactivity consistent with GFAP (**Figure 3E**), or 2) “GFAP-mimicking pattern”, which is a pattern atypical of GFAP but has liner or reticular reactivity. We also reviewed GFAP pattern on in-house IHC. GFAP-Ab were examined with established CBA at the laboratory of Josep Dalmau, when the patient had either GFAP or GFAP-mimicking pattern, or had clinico-radiologic features (such as radial periventricular enhancement on brain MRI) which were reported in patients with GFAP-positive meningoencephalomyelitis known as autoimmune GFAP astrocytopathy (18).

### 2.5 Evaluation of immunostaining pattern characteristic of individual NS antigens

In Part II, we evaluated an immunostaining pattern in NS-Ab-positive patients (n=85): NMDAR (n=60), LGI1 (n=9), GABAaR (n=8), AMPAR (n=3), GABAbR (n=3), Caspr2 (n=1), and GluK2 (n=1) (**Figure 1**).

In each pre-selected NS antigen, we characterized the immunostaining pattern at the level of the hippocampus and cerebellum, by focusing on the reactivity with NS antigens expressed on the following regions: dentate gyrus molecular layer (DG-ML), dentate hilus, cerebellar molecular layer (CB-ML), cerebellar granular layer (CB-GL), and cerebellar white matter (CB-WM), all of which are included in the commercial IHC biochips. We also paid attention to antigen-specific laminar reactivity in the DG-ML whether there is difference in intensity along the inner, middle, or outer molecular layers.

**Figure 4:**
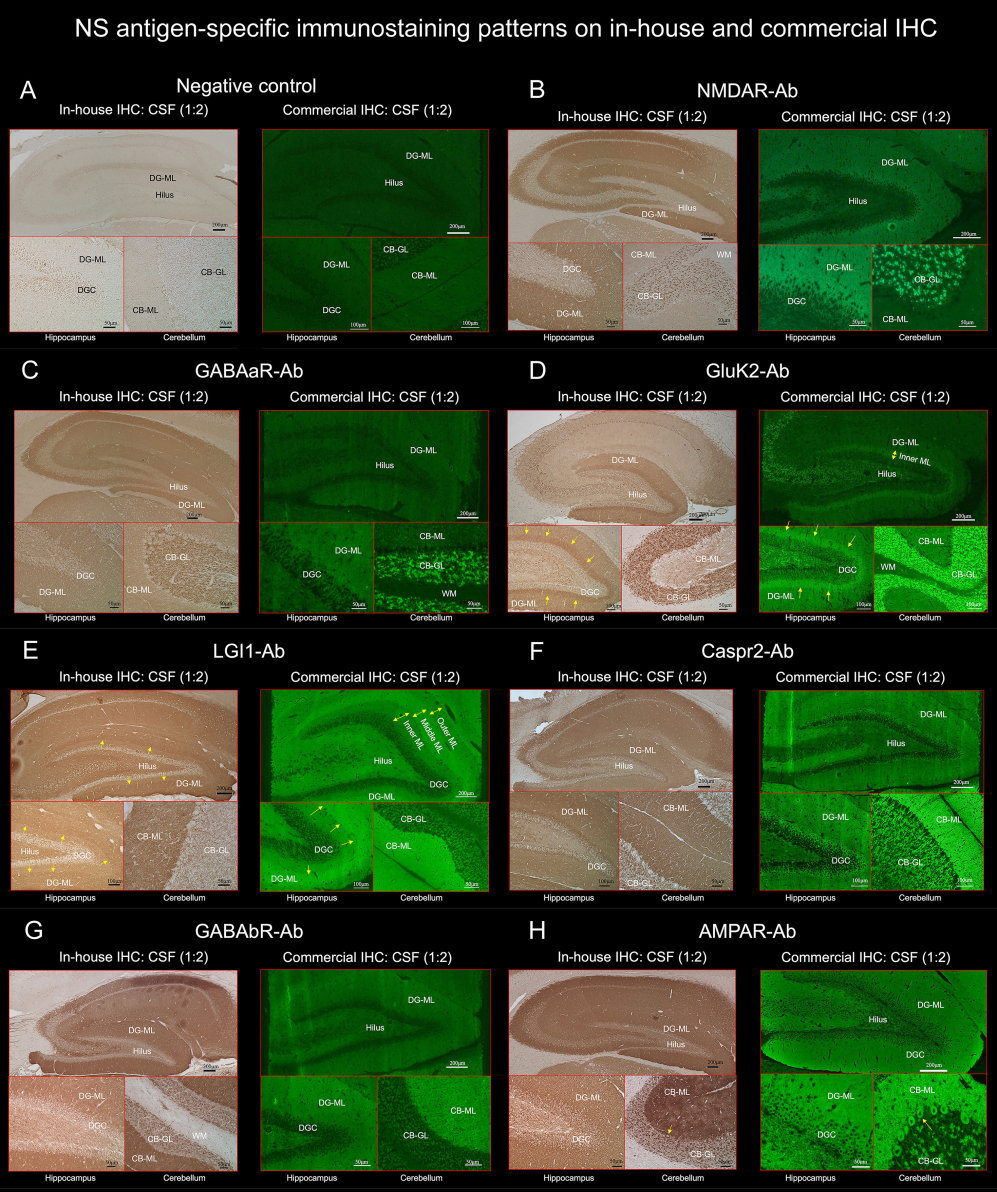
TBA reveals an immunostaining pattern highly characteristic of the individual NS antigen. (See Text). These preselected 7 NS antigens reveals intense reactivity with NS antigen “neuropil pattern”, among NS antigens, two reveal antigen-specific laminar reactivity, along the inner layer in GluK2 (**D**, arrows) and the middle layer in LGI1 (**E**, arrows). The first 3 NS antigens (NMDAR, GABAaR, GluK2) have dot-like reactivity on the CB-GL **(B–D)**, while the other 4 (LGI1, Caspr2, GABAbR, AMPAR) have homogenous reactivity on the CB-ML with some different immunoreactivity on the hilus, CB-GL, or Purkinje cells (see Text). CB-GL, cerebellar granular layer; CB-ML, cerebellar molecular layer; DGC, dentate granule cells; DG-ML, dentate gyrus molecular layer; ML, molecular layer; WM, white matter.

The immunostaining pattern characteristic of the individual NS antigens was determined mainly based on the results that showed a robust “neuropil pattern” on both in-house and commercial IHC (**Figures 1**, **4**) while excluding antibody-positive CSF samples with low titer to ensure a more accurate staining pattern. A series of illustrations was finally created by TI to make it easy to follow the difference of immunostaining pattern in each NS antigen using Adobe Illustrator (Adobe Inc.).

### 2.6 Standard protocol approvals, registrations, and patient consents

The study was approved by Institutional Review Boards of Kitasato University (B20-280). Written informed consent was obtained from the patients or their proxies. Information on symptoms, CSF, MRI, EEG, and treatments were obtained from the authors or referring physicians.

### 2.7 Statistical analysis

Statistical analyses were performed using JMP, version 14.2.0 (SAS Institute Inc.). The Fisher exact test was performed for comparison of categorical variables, and the Mann-Whitney test was used for continuous variables. The statistical significance was set at *p* < 0.05. The sensitivity and specificity of the commercial IHC were determined with 2-way contingency table analysis using a statistical calculator (MedCalc Software Ltd. https://www.medcalc.org/calc/diagnostic_test.php [Version 20.116]).

## 3 Results

### 3.1 Part I-1. Sensitivity and specificity of “neuropil pattern”

We found “neuropil pattern” in 67 of 261 patients (25.7%), while NS-Ab were identified in 97 patients (37.2%) (**Figure 1**). NS antigens identified in the CSF included NMDAR (n=62), LGI1 (n=11), GABAaR (n=8), GABAbR (n=5), AMPAR (n=4), GlyR (n=3), Caspr2 (n=2), GluK2 (n=1), and NS antigen not characterized yet (n=7); 5 patients had antibodies against more than one NS antigen. The “neuropil pattern” was seen in 64 of the 97 NS-Ab-positive patients (66.0%) but not in any of the 3 GlyR-Ab-positive patients. The “neuropil pattern” was seen in all 7 pre-selected major NS antigens as follows: GluK2 (1/1, 100%), Caspr2 (1/1, 100%), LGI1 (8/9, 88.9%), NMDAR (42/60, 70.0%), AMPAR (2/3, 66.7%), GABAbR (2/3, 66.7%), and GABAaR (3/8, 37.5%); the 5 patients with multiple NS-Ab were not included in the evaluation of frequency of the “neuropil pattern” in each NS antigen.

NS-Ab were more frequently identified in patients with “neuropil pattern” than those without (64/67 [95.5%] vs 33/194 [17.0%], *p* <.0001). Sensitivity and specificity of the “neuropil pattern” for predicting NS-Ab were 66.0% (95% CI 55.7-75.3), and 98.2% (95% CI 94.8-99.6), respectively. False-positive rate was 1.8% (3/164) while false-negative rate was 34.0% (33/97).

All 3 false-positive patients had high titers of GAD antibodies in their sera (> 2,000 U/mL, reference < 5), in all of them, the commercial IHC revealed neuropil-like reactivity in the DG-ML, but a pattern of synaptic staining in the CB-ML, CB-GL, and the axon hillocks of Purkinje cells (PC) which are highly characteristic of GAD65 (1) (**Figures 3A, B**). No NS-Ab were found in either serum or CSF in the 3 patients. We also confirmed the presence of GAD65-Ab in their CSF with GAD65-transfected CBA in the 3 patients. The neuropil-like staining was finally considered attributed to high titers of GAD65 antibodies. High titers of GAD antibodies were also found in the other 5 patients, without apparent “neuropil pattern” but a pattern of GAD65 reactivity was seen in the 5 patients.

Among the identified NS antigens in the false-negative patients (n=33), NMDAR was most frequently found (n=18, 54.5%), followed by GABAaR (n=5), GlyR (n=3, 1 with concurrent GABAbR), GABAbR (n=1), LGI1 (n=1), AMPAR (n=1), and not characterized yet (n=4).

To elucidate the factors potentially related to the false-negative results, we added in-house IHC in 3 false-negative patients with GABAaR-Ab (n=1), LGI1-Ab (n=1), or NMDAR-Ab (n=1). In the all 3 patients, in-house IHC revealed visually recognizable reactivity with corresponding NS antigens (**Figures 3C, D**). In patients with NMDAR-Ab, the frequency of low antibody titers (< 1:32) was higher in “neuropil-negative” patients than in “neuropil-positive” patients (16/18 [88.9%] vs 1/42 [2.4%]) (*p* <.0001). It was also noted that false-negative rate was higher in GABAaR than in LGI1 (5/8 [62.5%] vs 1/9 [11.1%]), *p* = 0.0498) but not significantly than in NMDAR (5/8 [62.5%] vs 18/60 [30.0%], *p* = 0.1088). However, antibody titers were not measured in GABAaR because no commercial CBA is available.

### 3.2 Part I-2. Clinical relevance of “GFAP pattern” on commercial IHC

Of 261 patients, 25 (9.6%) had either GFAP (n=21) or GFAP-mimicking pattern (n=4) (**Figure 1**). GFAP-Ab were examined in 31 patients including 6 who did not have either pattern but clinico-radiologic features reported in GFAP-positive meningoencephalomyelitis, and were identified in 21 patients (67.7%): 20 of the 21 patients (95.2%) with GFAP pattern, 1 of the 4 patients (25.0%) with GFAP-mimicking pattern, but not in any of the other 6 patients. Sensitivity and specificity of “GFAP pattern” for predicting GFAP-Ab were 95.2% (95% CI 76.2-99.9) and 90.0% (95% CI 55.5-99.8), respectively. The GFAP pattern was more clearly seen on commercial IHC than on in-house IHC (**Figure 3E**). In addition to GFAP pattern, the commercial IHC revealed intense reactivity along the cerebellar white matter, which was later confirmed due to concurrent MOG antibodies (**Figure 3F**).

In this cohort, the GFAP-Ab-positive patients were male predominant (13 male [61.9%]), with a median age at onset of 55 years (range, 18-79 years). Their main clinical features consisted of diverse phenotypes, including meningoencephalomyelitis (n=8), meningoencephalitis (n=3, 1 with diffuse sulcus enhancement), encephalitis (n=4, 1 with diffuse white matter MRI abnormalities, 1 with concurrent EBV DNA), myelitis (n=1), ADEM-like syndrome (n=2), stiff-person spectrum disorder-mimicking syndrome (n=1), encephalopathy (n=1), or overlapping anti-NMDAR encephalitis and MOG-positive demyelinating syndrome (n=1).

In one patient, GFAP-Ab were not identified in either serum or CSF despite the presence of apparent “GFAP pattern” on commercial IHC. The patient presented with acute onset of memory and psychobehavioral alterations associated with persistent fever of unknown etiology. CSF examination revealed mild pleocytosis (< 70 white blood cells/µL), without oligoclonal band detection. A brain MRI showed only mildly increased diffusion-weighted image/fluid-attenuated inversion recovery signal in the left insular cortex and ipsilateral fimbria, without abnormal enhancement, and an EEG revealed periodic lateralized epileptiform discharges in the left cerebral hemisphere, but no NS-Ab were identified in either serum or CSF. The clinico-radiologic features were not particularly suggestive of GFAP-Ab; however, GFAP-pattern was seen on commercial IHC (**Supplementary Figure 1**). In order to clarify whether the filamentous immunoreactivity is consistent with GFAP, we performed double immunolabeling of rat brain IHC using the patient’s antibodies and commercial GFAP monoclonal antibody (Clone GA5; Invitrogen), which revealed colocalization of reactivities, indicating that the patient’s IgG recognizes GFAP (**Supplementary Figure 1**).

### 3.3 Part II. Immunostaining patterns of individual NS antigens

TBA revealed an immunostaining pattern highly characteristic of each NS antigen on both commercial and in-house IHC in a similar distribution (**Figure 4**). TBA did not reveal apparent “neuropil pattern” in NS-Ab-negative CSF (**Figure 4A**). The immunostaining pattern of each NS antigen was as follows:

NMDAR revealed homogenous reactivity in the DG-ML with less intense dot-like reactivity in the CB-GL (**Figure 4B**). The regional intensity in the DG-ML was homogenous but more intensely stained in the surroundings of the dentate granule cells compared with the middle or outer molecular layer.

Both GABAaR and GluK2 revealed intense dot-like reactivity in the CB-GL (more densely stained in GluK2 than in GABAaR), but GABAaR revealed homogenous reactivity in the DG-ML (**Figure 4C**) while GluK2 revealed laminar reactivity in the DG-ML with strongest reactivity along the inner molecular layer (**Figure 4D**, arrows).

**Figure 5:**
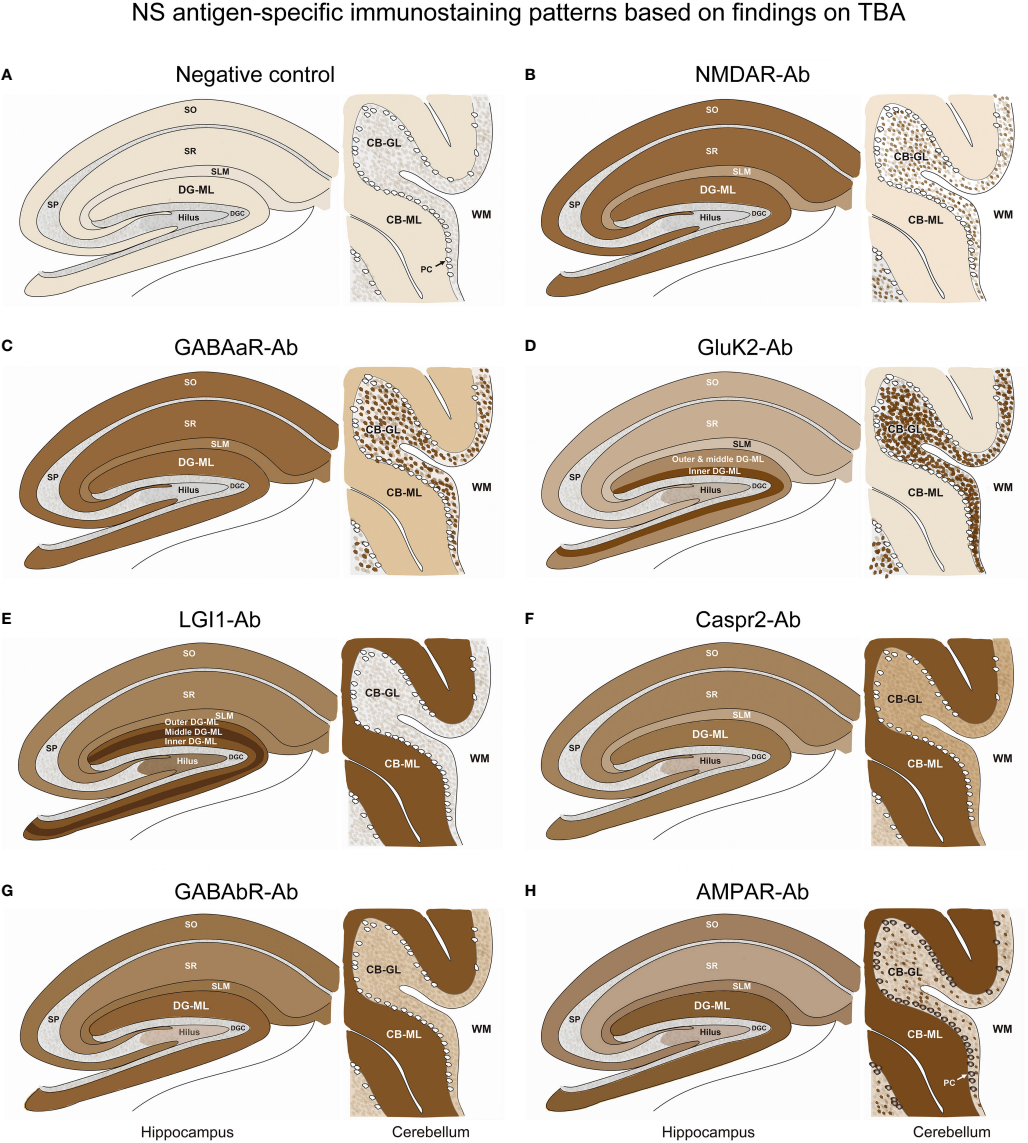
A series of illustrations are created as simple as possible to make it easy to follow individual staining pattern at the level of hippocampus and cerebellum. See Text and **Figure 4**. CB-GL, cerebellar granular layer; CB-ML, cerebellar molecular layer; DGC, dentate granule cells; DG-ML, dentate gyrus molecular layer; PC, Purkinje cells; SLM, stratum lacunosum moleculare; SO, stratum oriens; SP, stratum pyramidale; SR, stratum radiatum; WM, white matter.

LGI1, Caspr2, GABAbR, and AMPAR revealed intense homogenous reactivity in the CB-ML but LGI1 revealed laminar reactivity in the DG-ML with strongest reactivity along the middle layer, that is highly contrast to a pattern of GluK2 reactivity. Whereas Caspr2, GABAbR, and AMPAR revealed homogenous reactivity in the DG-ML. The dentate hilus was intensely stained in LGI1 but only mildly in Caspr2, GABAbR, and AMPAR (**Figures 4E–H**). The CB-GL was not apparently stained in LGI1 but mild to moderately as a reticular pattern in Caspr2 and GABAbR, and mild to moderately as a dot-like pattern in AMPAR; there was intense reactivity with cytoplasmic protein in the PC (**Figure 4H**, arrows) in 1 of the 3 patients with AMPAR antibodies with “neuropil pattern” but no reactivity with the PC in patients with other NS-Ab. Distinctive staining patterns were noted between NS antigens, but Caspr2 and GABAbR revealed much similar staining pattern (**Figures 4**, **5**, and **Table 1**).

**Table 1:** Immunostaining pattern highly characteristic of major neuronal surface antigens.

NS antigen	DG-ML	Dentate hilus	CB-ML	CB-GL	CB-WM	Purkinje cells
NMDAR	Homogenous	None	None	Dot-like, mild	None	None
GABAaR	Homogenous	None to mild	Mild	Dot-like, moderate to intense	None	None
GluK2	Laminar: inner layer > middle or outer layer	Mild	Mild	Dot-like, intense	None	None
LGI1	Laminar: middle layer > inner or outer layer	Intense	Intense	Reticular, very mild	None	None
Caspr2	Homogenous	Mild	Intense	Reticular, mild to moderate	None	None
GABAbR	Homogenous	Mild	Intense	Reticular, mild to moderate	None	None
AMPAR	Homogenous	Mild	Intense	Dot-like, mild to moderate	None	Cytoplasmic but not always

CB-GL, cerebellar granular layer; CB-ML, cerebellar molecular layer; CB-WM, cerebellar white matter; DG-ML, dentate gyrus molecular layer.

## 4 Discussion

This study reveals that 1) the commercial IHC is useful for screening NS-Ab, but the negative results should be interpreted cautiously because the “neuropil pattern” may be missed in up to one-thirds of the NS-Ab-positive patients particularly when antibody titers are low or due to other reasons, 2) the commercial IHC also provides an easily recognizable “GFAP pattern”, and 3) this study confirmed the previous observation that individual NS antigens have antigen-specific immunostaining pattern, which can be used as a biomarker to estimate the target NS antigen.

TBA has been used to identify autoantibodies against not only classical paraneoplastic neuronal (intracellular) antigens but also NS antigens (synaptic receptors or other membrane proteins) (1, 2). However, the brain tissue processing is quite different between intracellular and cell-surface antigens (16, 19, 20). To detect NS-Ab, a tissue processing adapted to cell surface or synaptic proteins is required to preserve the native conformation of the target antigen (1). Tissue perfusion should not be performed before decapitation, and it is recommended that the non-perfused brains are fixed in 4% paraformaldehyde for 1 hour at 4°C (16), as described above. Such IHC has mainly been used at research laboratories. NS-Ab is known to produce “neuropil pattern”, but individual staining pattern has not been extensively studied. Therefore, we conducted this study.

Sensitivity and specificity of the “neuropil pattern” identified on commercial IHC for predicting NS-Ab were 66.0% and 98.2%, respectively. All 3 false-positive patients had high titers of GAD65 antibodies, thus, the neuropil-like pattern seen on TBA was considered to reflect abundant GAD65 immunoreactivity in the axon terminals (21). GAD-Ab are easily examined with ELISA or EIA and show a synaptic pattern characteristic of GAD65. Therefore, such mild reactivity mimicking “neuropil pattern” is less likely to cause confusion in the diagnosis of AE or related disorder. However, relatively high false-negative rate (34.0%) cannot be ignored in clinical practice.

To evaluate factors involved in the false-negative results, we added in-house IHC. In at least 3 false-negative patients, the “neuropil pattern” was visually identified on our in-house IHC, suggesting that the “neuropil pattern” may be more clearly shown in our in-house IHC adapted to NS antigen compared with the commercial IHC, of which tissue processing is not available due to industrial secrets, thus we do not know whether the commercial IHC is adapted to NS antigen or not. Further study will be required to determine which one is better in terms of detection of “neuropil pattern”.

To address the issue whether antibody titers influence detection rate of “neuropil pattern”, we measured antibody titers in 60 patients with anti-NMDAR encephalitis because NMDAR consisted of 54.5% of NS antigens identified in the false-negative patients. As expected, low antibody titers (< 1:32) were more frequently found in false-negative patients compared with “neuropil pattern”-positive patients, suggesting that low antibody titers may be one of the major causes of the false-negative results at least in NMDAR-Ab. Thus, it should be kept in mind that antibody titers may influence the detection rate of the “neuropil pattern” on commercial IHC.

It is also important to note that the detection rate of “neuropil pattern” was different among NS antigens. Although we did not measure antibody titers in NS antigens other than NMDAR, the detection rate may be lower in GABAaR than in other NS antigens. At this point, the cause of higher false-negative results in GABAaR compared with other NS antigen remains speculative. We did not measure antibody titers; thus, we cannot exclude a potential effect of low antibody titers on the higher false-negative results; however, we also concern about a technical difficulty during tissue preparation; we have sometimes faced a difficulty in revealing “neuropil pattern” in GABAaR-Ab-positive samples, even with in-house IHC adapted to NS antigen. It seems to be more difficult to preserve the native conformation of the epitope in GABAaR compared with other NS antigens (such as NMDAR or LGI1). Further studies are required to conclude whether the detection rate is different among NS antigens.

In Part I, we also evaluated a predictive value of “GFAP pattern”. When GFAP-Ab are positive, IHC revealed reactivity along the radial glia of Bergmann in the CB-ML and astrocytes in the CB-GL. Although the number of patients examined with CBA is quite small, most of the patients with a pattern of GFAP reactivity were confirmed to have GFAP-Ab in their CSF. Sensitivity and specificity of the “GFAP pattern” were 95.2% and 90.0%, respectively, and a cactus thorn-like or filamentous staining is easily recognizable on commercial IHC. Although “GFAP pattern” highly suggests the presence of GFAP-Ab, IgG GFAP-Ab were not identified with CBA in either serum or CSF in one of the 21 “GFAP pattern”-positive patients.

The reason why GFAP-Ab are not identified with CBA despite the presence of “GFAP pattern” is unclear in the patient, but the discrepancy between commercial IHC and CBA may be explained by difference of GFAP isoform that the patient’s IgG antibodies recognize. GFAP is an intermediate filament protein expressed in astrocytes, and has 10 different GFAP isoforms. Among those, GFAP α and δ/ε are the main isoforms (22). After the discovery of GFAP-Ab in 2016 (18), GFAP-α isoform-transfected CBA has been used as a standard test for antibodies against GFAP (23). Therefore, we performed double immunolabeling of rat brain IHC using the patient’s antibodies and commercial GFAP monoclonal antibody, which revealed colocalization of reactivities (**Supplementary Figure 1**). A previous study (24) reported that some of the GFAP-Ab-positive patients may be positive for antibodies against only GFAP δ/ε isoform; therefore, the false-positive results may be due to IgG autoantibodies against GFAP isoform other than GFAP α isoform. Immunoreactivity discrepancies observed between recombinant GFAP proteins and rodent brain tissue has also been reported, and possible explanations have been discussed, including posttranslational modification of GFAP-α antigenicity or obscuring of a dominant GFAP-α epitope in tissue by 3-dimensional *in situ* interaction between GFAP isoforms and other intermediate filament proteins (18).

Although GFAP-Ab were identified in 21 of 31 examined patients, GFAP is an intracellular autoantigen, thus the IgG GFAP-Ab is unlikely pathogenic (1). GFAP-positive meningoencephalitis is currently presumed to be T-cell mediated (25), and the antibodies can be seen without association of distinct clinical-radiological features (3), but the presence of IgG GFAP-Ab provides a clue supporting immune-mediated disorder as a biomarker of the disease.

In Part II, we characterized NS-antigen-specific immunostaining patterns, and created a series of illustrations by simplifying their staining patterns to make it easy for physicians or observers to recognize antigen-specific pattern (**Figure 5**). A pattern recognition is important in estimation of NS antigen based on the IHC. Based on the results, we grossly divided the staining pattern into 2 groups; one is a pattern of CB-GL predominant reactivity characterized by a dot-like staining in the CB-GL and the other is a pattern of CB-ML predominant reactivity characterized by homogenous intense staining in the CB-ML as a common characteristic feature. The NMDAR, GABAaR, and GluK2 are included in the former group, while the LGI1, Caspr2, GABAbR, and AMPAR are in the latter one.

The remarkable finding is that this study revealed an antigen-specific laminar reactivity in the DG-ML; strongest reactivity was seen along the middle layer in LGI1 while along the inner layer in GluK2. These patterns are easily recognizable on commercial IHC than on in-house IHC and can be used as a diagnostic marker of NS antigen.

LGI1, Caspr2, GABAbR, and AMPAR show similar intense reactivity in the CB-ML, but there are some different reactivities in the other regions. The dentate hilus is intensely stained in LGI1, but only mildly in Caspr2, GABAaR, GluK2, and GABAbR, or not in NMDAR. Cytoplasmic antigen in the PC may be stained in AMPAR, but not in the other 6 NS antigens. Cytoplasmic staining of the PC may be non-specific and may not be always seen even in AMPAR; however, the cytoplasmic staining of PC has been described (7), and AMPAR subunits are shown to be expressed by the PC (26). Thus, the presence of apparent PC staining sparing the nucleus, with intense reactivity in the CB-ML and sparse but dot-like staining in the CB-GL may suggest AMPAR.

Intensity of the immunoreactivity is more likely to reflect high antibody titers as well as preservation of the native conformation of the epitope, while its distribution is more likely to reflect the location of the target NS antigen expressed in the rodent brain tissue. Although exact distribution of the individual NS antigens has not been well characterized yet in a rodent brain, the intense reactivity along the inner layer of the DG-ML is consistent with expression of GluK2 (27). The dot-like immunoreactivity in the CB-GL seen in NMDAR, GABAaR, GluK2, and AMPAR is presumed due to the reactivity with corresponding NS antigen in the cerebellar glomeruli, which are an intertwined cluster of nerve fibers surrounded by glia where mossy fibers synapse with granule cell axons, where NMDAR, GABAaR, GluK2 and AMPAR are all expressed on cell surface membrane (28–31). Clarification of the distribution of each NS antigen may help identification of novel antibodies against yet unknown antigen.

This study has limitations; no detailed information is available about the brain tissue processing in commercial IHC; antibody titer was determined for NMDAR antibodies with commercial fixed CBA but not with live CBA, and not determined for other NS antigens; the number of patients examined for GFAP-Ab is small; the number of patients examined for immunostaining pattern is also small (1 or 3) in each group of the NS antigen other than NMDAR; immunostaining pattern examined with serum is not taken into account in the analysis for immunostaining pattern; the predictive value of the commercial IHC or in-house IHC is not assessed in a prospective manner; and immunostaining pattern was assessed for preselected 7 major NS antigens, but not for other NS antigens identified to date.

Despite these limitations, this study demonstrates that both in-house and commercial IHC are clinically useful not only for screening NS- and GFAP-Ab, but also for estimating NS antigen. However, the results of the commercial IHC should be interpreted with caution while taking these limitations into consideration when predicting the presence of NS-Ab or estimating NS antigen. The strategy of the diagnosis of AE should be based on both clinical information and immunostaining pattern on TBA. The immunostaining pattern highly characteristic of NS antigens can be used as a biomarker of AE.

## Data availability statement

The original contributions presented in the study are included in article/**Supplementary Material**. Further inquiries can be directed to the corresponding author.

## Ethics statement

The studies involving human participants were reviewed and approved by Institutional Review Boards of Kitasato University (B20-280). Written informed consent to participate in this study was provided by the participants’ legal guardian/next of kin. The animal study was reviewed and approved by the Animal Experimentation and Ethics Committee of the Kitasato University School of Medicine (2022-031).

## Author contributions

NN and NK contributed to the drafting of the manuscript, the acquisition of major data of antibody assay using immunohistochemistry of the rodent brain, and the preparation of the figures. TM, MI, MNag, MNak, JK, EK, and KN contributed to the acquisition of clinical data. TI contributed to analysis of data, the acquisition of clinical data, the drafting of a significant portion of manuscript, the preparation of the figures including illustrations. All translations into English were done by the authors. All authors contributed to the article and approved the submitted version.
