# Supplementary material for: Neuronal surface antigen-specific immunostaining pattern on a rat brain immunohistochemistry in autoimmune encephalitis
Source: Front Immunol. 2023 Jan 16;13:1066830. doi: 10.3389/fimmu.2022.1066830 (PMC9885155; doi:10.3389/fimmu.2022.1066830)
Supplement: Supplementary file 1 [file DataSheet_1.docx]

Supplementary Material

# Supplementary Figure 1: Reactivity of the patient’s antibodies with hippocampus and colocalization with the commercial GFAP monoclonal antibodies


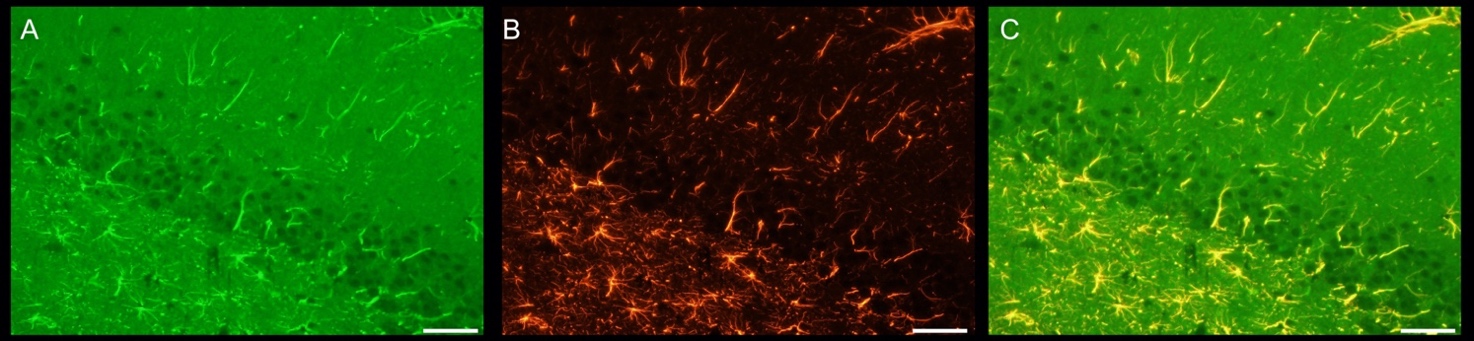


The patient’s CSF reveals a pattern of GFAP reactivity on a rat brain commercial IHC (A), but the CSF was reportedly negative for GFAP antibodies, which were examined with established CBA at the laboratory of Josep Dalmau. Therefore, we performed double immunolabeling using the patient’s antibodies (A, green) and commercial GFAP monoclonal antibody (B, red, Clone GA5 Invitrogen), which revealed colocalization of reactivities (C, yellow), indicating that the patient’s IgG recognize GFAP.

A scale bar = 50µm

CBA, cell-based assay; GFAP, glial fibrillary acidic protein; IHC, immunohistochemistry
